# The sensitivity of murine spermiogenesis to miglustat is a quantitative trait: a pharmacogenetic study

**DOI:** 10.1186/1477-7827-5-1

**Published:** 2007-01-22

**Authors:** Wilhelm Bone, Charlotte M Walden, Martin Fritsch, Ulrike Voigtmann, Eckhard Leifke, Ulrich Gottwald, Stephanie Boomkamp, Frances M Platt, Aarnoud C van der Spoel

**Affiliations:** 1 Schering AG, Müllerstr. 178, 13342 Berlin, Germany; 2 The Glycobiology Institute, Department of Biochemistry, University of Oxford, South Parks Road, Oxford OX1 3QU, UK; 3 Department of Pathology, University of Oxford, South Parks Road, Oxford OX1 3RE, UK; 4 Department of Pharmacology, University of Oxford, Mansfield Road, Oxford OX1 3QT, UK

## Abstract

**Background:**

A major event in the post-meiotic development of male germ cells is the formation of the acrosome. This process can be perturbed in C57BL/6 mice by administration of the small molecule miglustat (N-butyldeoxynojirimycin, NB-DNJ). The miglustat-treated mice produce morphologically abnormal spermatozoa that lack acrosomes and are poorly motile. In C57BL/6 mice, miglustat can be used to maintain long-term reversible infertility. In contrast, when miglustat was evaluated in normal men, it did not affect spermatogenesis. To gain more insight into this species difference we have now evaluated the reproductive effects of miglustat in rabbits, in multiple mouse strains and in interstrain hybrid mice.

**Methods:**

Male mice of 18 inbred strains were administered miglustat orally or via miniosmotic pumps. Rabbits were given the compound in their food. Fourth-generation interstrain hybrid mice, bred from C57BL/6 and FVB/N mice (which differ in their response to miglustat), also received the drug. Data on fertility (natural mating), sperm motility and morphology, acrosome status, and serum drug levels were collected.

**Results:**

In rabbits the drug did not induce aberrations of sperm shape or motility, although the serum level of miglustat in rabbits far exceeded the level in C57BL/6 mice (8.4 μM and 0.5 μM, respectively). In some strains of the Swiss and Castle lineages of inbred mice miglustat did not cause infertility, severe morphological sperm aberrations or reduced sperm motility. In these strains miglustat only had milder effects. However, miglustat strongly disturbed acrosome and sperm nucleus development in AKR/J and BALB/c mice and in a number of C57BL/6-related strains. The consequences of drug administration in the interstrain hybrid mice were highly variable. Judging by the number of grossly abnormal spermatozoa, these genetically heterogeneous mice displayed a continuous range of intermediate responses, distinct from either of their parental strains.

**Conclusion:**

The effects of miglustat on spermatogenesis in mice are strain-dependent, while in rabbits the drug is ineffective. Evaluation of interstrain hybrid mice indicated that the sensitivity of spermatogenesis to miglustat is a quantitative trait. These studies pave the way for identifying the genetic factors underlying the strain/species differences in the effect of miglustat.

## Background

In 2002 and 2003 miglustat was approved as an orphan drug by the Federal Drug Administration (USA) and the European Medicines Evaluation Agency, respectively, for the treatment of type 1 Gaucher disease, a lysosomal glycosphingolipid storage disorder. Miglustat can partially inhibit the biosynthesis of glucosylceramide, by inhibiting ceramide glucosyltransferase, and therefore can be used to reduce the glucosphingolipid levels in cells [1]. Miglustat is tolerated in humans and at high doses in mouse disease models [2,3]. Apart from the ceramide-specific glucosyltransferase miglustat inhibits *in vitro *a number of other enzymes: α-glucosidases I and II, sucrase, maltase, lysosomal glucocerebrosidase and the non-lysosomal glucosylceramidase [4-7]. The pharmacokinetics of miglustat are relatively uncomplicated with no or very limited metabolism, no protein binding and excretion predominantly into the urine [8]. Renal clearance in mouse and rat was greater than creatinine clearance, consistent with active renal secretion not seen in rhesus monkey, dog or man [9].

Three weeks of oral administration of miglustat to male C57BL/6 mice are sufficient to render them infertile [10]. The miglustat-induced infertility can be maintained for several months without giving rise to overt adverse effects, either physiological or behavioural [11]. Full fertility is regained when the drug is withdrawn, even after 12 months of administration [10,11]. Spermatozoa released from the epididymis of miglustat-treated C57BL/6 males display a spectrum of abnormal head shapes. Acrosomal antigens are mostly absent or display irregular patterns. In addition, the mitochondria of these cells often have an aberrant morphology and are arranged in relatively short and wide mitochondrial sheaths. The motility of the affected spermatozoa is severely impaired. ICSI experiments with misshapen spermatozoa from treated C57BL/6 mice showed that miglustat does affect sperm morphology and physiology, but does not diminish the genetic potential of spermatozoa [12].

The advanced clinical status of miglustat allowed the compound to be evaluated for its reproductive effects in a small number of normal healthy men. They received 100 mg of miglustat twice daily (on average 2 mg/kg/day) for 6 weeks, a similar dose as is given to Gaucher patients [13]. During drug treatment and the following 12 weeks various sperm parameters, including morphology and capacity to undergo the acrosome reaction were measured. In the miglustat-treated men none of the reproductive features were significantly affected. The question is now what underlies the difference in the response to miglustat between men and male C57BL/6 mice. We have assessed the effects of miglustat on sperm morphology, particularly shape of nucleus and acrosome, in rabbits and in 18 additional mouse strains. We have found large differences in the sensitivity to the drug between mouse strains, and also found the rabbit to be insensitive. This suggests that the susceptibility to miglustat has a genetic basis. We have therefore investigated its mode of inheritance by analyzing the effects of miglustat on sperm morphology in interstrain hybrid mice that were generated from two strains of mice that differ profoundly in their response to the drug.

## Methods

### Animals

Mice of the following inbred strains were purchased from Harlan UK (Bicester, Oxfordshire, UK): A/J, BALB/c, C3H/HeN, CBA/Ca, C57BL/6J, C57BL/10ScSn, DBA/2, FVB/N, MRL/Mp, NZB and NZW. Male mice of the 129S1/SvImJ, AKR/J, C57BR/J, C57L/J, C58, MA/MyJ, SM/J and YBR/J strains were purchased from The Jackson Laboratory (Bar Harbor, ME, USA). For the serum level experiments in mice C57BL/6J and FVB/N mice were purchased from Charles River Wiga GmbH (Sulzfeld, Germany).

Russenkaninchen rabbits strain (Crl:CHBB (HM)) were from Charles River Wiga GmbH (Sulzfeld, Germany). Male (2.4 – 3.2 kg, ca. 50 weeks) and female rabbits (2.6 – 3.0 kg,) were housed under 12 h light : 12 h dark (lights on 7:00 h) at 22°C and had access to standard chow (Ssniff, Soest, Germany) and water ad libitum., Only one strain of rabbits was used because of the high costs of miglustat. Also for this reason the Russenkaninchen strain was used because of the relatively small size of the animals of this strain.

All animals were housed and sacrificed in accordance with the UK Home Office Animals (Scientific Procedures) Act 1986 or in accordance with German legal requirements dependent on the place where experiments have been performed.

### Fertility in rabbits

After passing a predosing phase of at least 2 weeks, 5 male rabbits were fed 5, 15 or 50 mg/kg/day miglustat (Toronto Research Chemicals, Toronto, Canada) for 8 weeks. Miglustat was added to the standard chow and pressed into pellets (Ssniff, Soest, Germany). Average drug uptake was 5.2 ± 0.6 mg/kg/day in the 5 mg/kg/day group, 15.3 ± 2.0 mg/kg/day in the 15 mg/kg/day group and 49.1 ± 3.8 mg/kg/day in the 50 mg/kg/day group. Control animals received standard chow without miglustat. Food intake was not affected by the presence of miglustat in the chow. Semen and blood samples were taken every 2 weeks. Blood was taken from the ear artery for measuring miglustat levels with LC/MS. Semen samples were collected according to [14]. Eight weeks after beginning the treatment male rabbits of the control group and the 50 mg/kg/day group were caged together with one female each for determination of fertility.

### Quality of semen/spermatozoa from rabbits

Semen parameters determined were as follows: volume, colour, pH, coagulation and sperm count. Spermatozoa were subjected to computer-assisted sperm analysis (CASA) (Hamilton Thorne Research, Beverly, MA, USA). Percentage motile spermatozoa was estimated manually by counting flagellating or non flagellating spermatozoa. For the analysis by CASA 10 minutes after collection spermatozoa were diluted 20-times in medium (NaCl 97 mM, KCl 3 mM, Na_2_HPO_4 _2.8 mM, MgCl_2 _4 mM, CaCl_2 _2 mM, Na-pyruvate 0.2 mM, glucose 5 mM, HEPES 10 mM, BSA 6 g/l, NaHCO_3 _25 mM, lactate 32.8 mM) and approximately 10 μl of the suspension were placed in a 20 μm deep sperm analysis chamber at 37°C (Standard Count Analysis Chambers, Leja, Nieuw-Vennep, Netherlands). Ten digitised film sequences were recorded (60 Hz). In general, 100 sperm tracks from the recording were analysed by CASA (Animal Motility, version 12.2). The settings used were as follows: frame rate: 60 Hz, number of frames analysed: 30, minimum contrast: 50, minimum size: 7, minimum number of detected data points for a track: 10. For counting the number of spermatozoa with aberrations air-dried sperm smears were made and stained with PNA/DAPI (lectin PNA from Arachis hypogaea, Alexa Fluor 488 conjugate, Molecular Probes, Oregon, USA and 4',6-Diamidino-2-phenylindole Dihydrochloride, Sigma Aldrich, Germany diluted in PBS (GIBCO)).

### Drug administration and mating tests in mice

Miglustat (gift from Oxford GlycoSciences, Abingdon, UK/CellTech UK, Slough, Berkshire, UK, or purchased from Toronto Research Chemicals, Toronto, Canada), as a dry crystalline solid powder, was mixed thoroughly with powdered mouse chow (expanded rat and mouse chow 1, SDS, Witham, Essex, U.K.) and stored at room temperature. Male mice were fed powdered mouse chow with or without miglustat for 5 weeks, at indicated doses. Alternatively, the drug was administered via a mini-osmotic pump (Model 2004, pump rate 0.22 – 0.26 μl/h, Alzet, DURECT Corporation, California, USA) for 7 weeks. The mice were 6 weeks old at the start of miglustat treatment. Natural mating tests were performed as described by van der Spoel *et al *[10]. Briefly, each male mouse was caged with two or four female 10-week old C57BL/6 mice for nine days, after which the male was removed and the females were monitored for pregnancies and litter sizes.

### Fluorescence microscopy of murine spermatozoa

Cauda epididymides were dissociated in M2 medium (Sigma) with forceps. Smears of spermatozoa were prepared as described by van der Spoel [10] and stained by indirect immunofluorescence with monoclonal antibody Mab18.6 [15], and Alexa488-conjugated goat anti-mouse IgG (Molecular Probes). Propidium iodide (1 μg/ml) was used as a nuclear stain. All antibodies were diluted in PBS containing 0.5% (w/v) BSA and 0.15 M glycine. Nuclear morphology and acrosomal staining were assessed using a Zeiss Axioskop 2 plus microscope. Images were acquired with a Zeiss 510 META confocal fluorescence instrument.

### Western blotting

Mouse spermatozoa were released by gentle pressure from caudae epididymides into ice-cold PBS containing Ca^2+ ^and Mg^2+^, and washed twice in cold 10 mM Tris-HCl, pH 8.0, 1 mM EDTA, and taken up in Laemmli sample buffer. Aliquots of lysed cells containing 5 μg protein were separated by SDS-PAGE and transferred to Immobilon-P polyvinylidene fluoride membranes (Millipore, Watford, UK). Blots were blocked in 10% milk powder in Tris-buffered saline containing 0.05% Tween-20 (TBST), and incubated with affinity-purified anti-bovine IAM38 antibodies [16] (1:1000, overnight) or affinity-purified anti-VYK antibodies [17] (1.2 ng/ml, overnight) in 2% milk powder in TBST. Anti-VYK is an antiserum raised against a synthetic peptide corresponding to residues 542–558 from murine sp56 [17]. Anti-human cytochrome C oxidase subunit I (A6403, Molecular Probes) was used (1:500, 2 hr) to verify equal loading. Blots were washed in TBST, incubated with horse-radish peroxidase-conjugated anti-IgG antibodies (Vector Laboratories, Peterborough, UK), washed, and developed with a chemiluminescent substrate (ECL Advance Western Blotting Detection Kit; Amersham, Chalfont St.Giles, Buckinghamshire, UK).

### Serum levels of miglustat

Serum level of miglustat was determined using liquid chromatography and mass spectrometry. For sample preparation acetonitrile (4-times the sample volume) including an internal standard (1 μM N-(4-Chlorophenyl)-2-[(4-pyridylmethyl)amino]benzamide) was added to the serum sample and mixed thoroughly. Samples were spun for 20 min at 4°C and > 2000 × g. The supernatant was removed by rotary evaporation and reconstituted with 20% acetonitrile solution. HPLC was performed on a Varian Polaris C18-A column (particle size: 3 μm, column dimensions: 2 × 50 mm, Varian, Palo Alto, CA, USA) at a flow rate of 0.3 ml/min. Eluents were 0.025% NH_3_·H_2_O in water with an increasing acetonitrile gradient (5–95% over 3 min). Eluates were analysed by tandem mass spectrometry.

### Statistics

Quantitative data were statistically evaluated with Student's t-test (comparison between two groups) or one-way ANOVA with Tukey's post-hoc test (more than two groups) using GRAPHPAD INSTAT version 3.0a for Macintosh (GraphPad Software). Values were considered statistically significantly different when *P *< 0.05.

## Results

### Miglustat has no effect on fertility in male rabbits

Miglustat uptake did not affect the bodyweight and food intake of the rabbits (data not shown). Each of the 5 male rabbits in the control group and in the 50 mg/kg/day group were mated to one female each after 8 weeks of miglustat treatment. All of the 5 drug-treated rabbits were fertile, comparable to the rabbits of the control group (Table 1). Also, the litter size was not affected by miglustat treatment (Table 1).

**Table 1:** Effects of miglustat administration on the fertility of male mice and rabbits as assessed by natural mating.

Species	Strain	Pregnancies/male		Pups/litter	
		Control	Miglustat	Control	Miglustat

Rabbit	Russen-Kaninchen	1.0 ± 0.0	1.0 ± 0.0	7.0 ± 1.2	7.2 ± 1.5
Mouse	C57BL/6	2.6 ± 0.9	0.0 ± 0.0*	6.7 ± 1.0	N/A
	129S1/SvImJ	1.6 ± 0.5	1.6 ± 0.5	8.2 ± 0.6	8.9 ± 1.9
	FVB/N	2.6 ± 1.5	2.2 ± 1.5	6.7 ± 1.6	6.0 ± 0.7
	DBA/2	3.0 ± 0.7	2.8 ± 1.1	7.5 ± 0.8	6.3 ± 1.3
	AKR/J	1.8 ± 0.4	1.4 ± 0.5	6.2 ± 2.3	3.6 ± 1.5
	MA/MyJ	1.6 ± 0.5	0.8 ± 0.8	7.8 ± 0.8	3.8 ± 3.3

Data were obtained from 5 males/5 × 1 female (rabbits), from 5 males/5 × 4 females per strain (C57BL/6, FVB/N and DBA/2), or from 5 males/5 × 2 females per strain (129S1/SvImJ, AKR/J and MA/MyJ). Miglustat administration to rabbits and mice was at 50 and 150 mg/kg/day, respectively. Data are expressed as means ± SD. Only the value marked with (*) is statistically different from control (*P *< 0.0002).

### Sperm morphology and motility in rabbits

**Figure 1:**
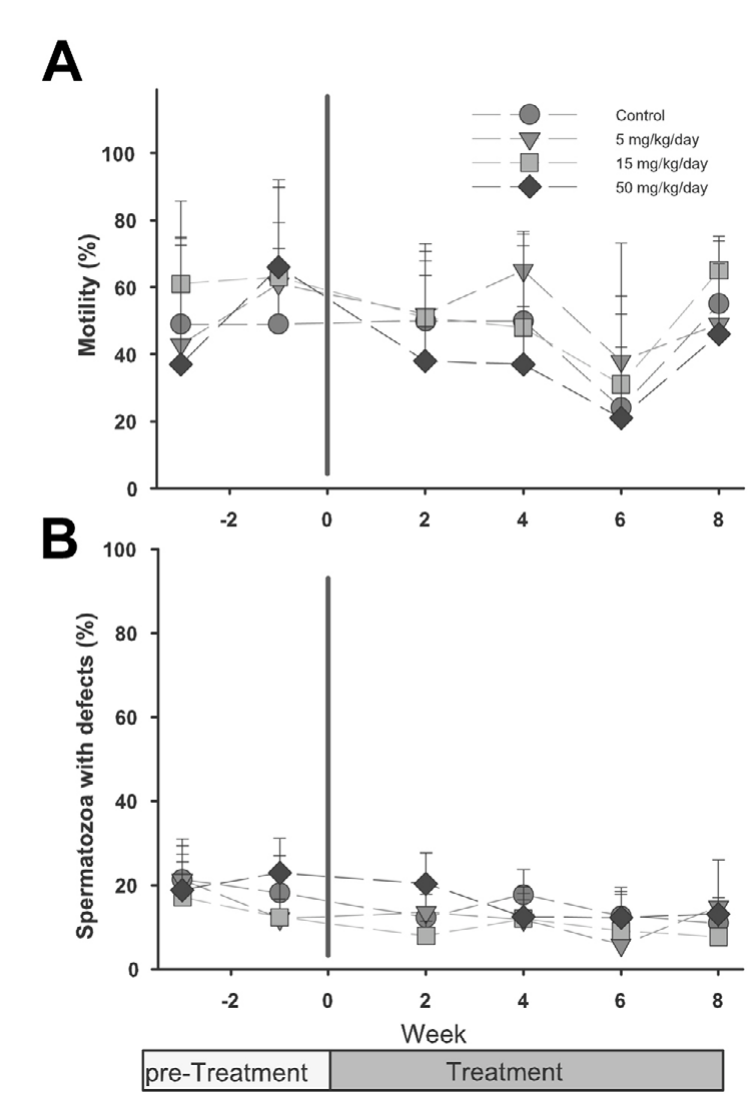
**Sperm motility (%) and morphology in rabbits**. *A*. Spermatozoa were released in medium and percentage motile spermatozoa was estimated by counting flagellating or non flagellating spermatozoa. *B*. For assessment of morphology, a smear was air-dried on a slide and stained with PNA and DAPI and counted manually for abnormal sperm heads. Data are presented as the mean ± SD of percentage motile spermatozoa for 5 males and as the mean ± SD of percentage abnormal spermatozoa. Nuclear morphology and acrosomal staining were assessed by examining 100 spermatozoa per rabbit.

**Figure 2:**
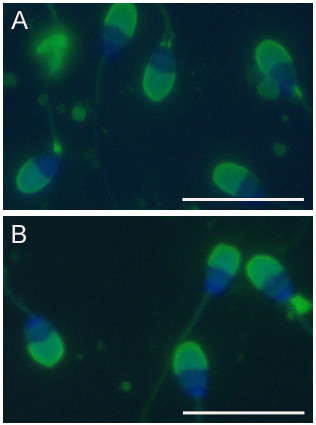
**Morphology of rabbit spermatozoa**. Smears of ejaculated spermatozoa were air-dried and stained with PNA/DAPI. *A*. Spermatozoa from control rabbits. *B*. Spermatozoa from rabbits treated with 50 mg/kg/day miglustat for 8 weeks. Bar = 20 μm.

Coagulation, pH, colour, volume of semen and sperm count remained unchanged at all the doses of miglustat that have been tested (data not shown). Furthermore, analysis of kinematic parameters by CASA did not reveal any significant change (percentage motile spermatozoa in Fig. 1A). In order to compare with the most prominent effect of miglustat in male C57BL/6 mice, the number of spermatozoa lacking intact acrosomes was counted using a PNA/DAPI staining method (Fig. 2). The percentage of spermatozoa that lacked intact acrosomes remained stable at approximately 20% in all doses for 8 weeks of treatment (Fig. 1B).

### Sperm morphology and motility in multiple mouse strains

**Figure 3:**
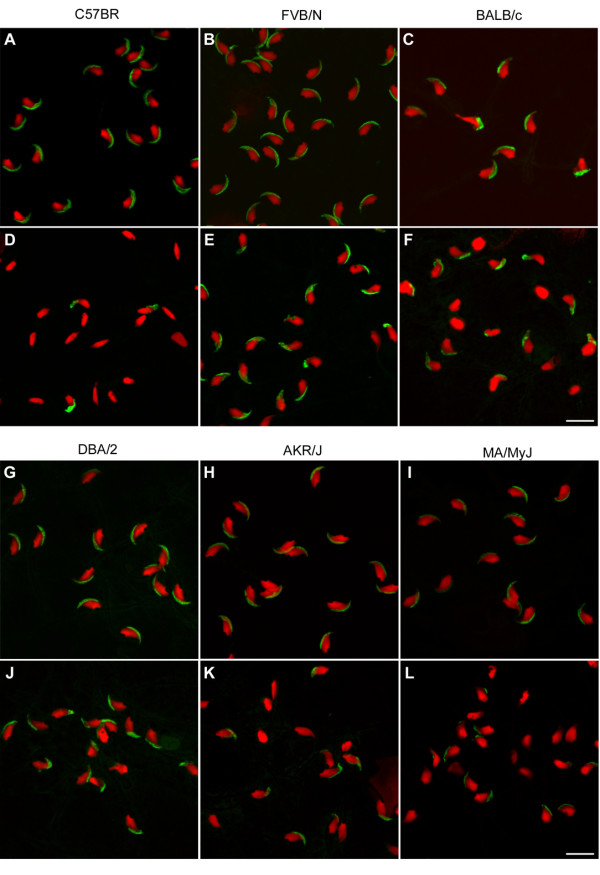
**Effects of miglustat on morphology of spermatozoa from various mouse strains**. Acrosomal and nuclear morphology of cauda epididymal spermatozoa from (*A*, *B*, *C*, *G*, *H *and *I*) control and (*D*, *E*, *F*, *J*, *K *and *L*) miglustat-treated mice from inbred mouse strains, (*A *and *D*) C57BR, (*B *and *E*) FVB/N, (*C *and *F*) BALB/c, (*G *and *J*) DBA/2, (*H *and *K*) AKR/J and (*I *and *L*) MA/MyJ. Acrosomes were stained with the monoclonal antibody Mab18.6 (green) and nuclei with propidium iodide (red). Drug administration was at 150 mg/kg/day. Bar = 10 μm.

**Figure 4:**
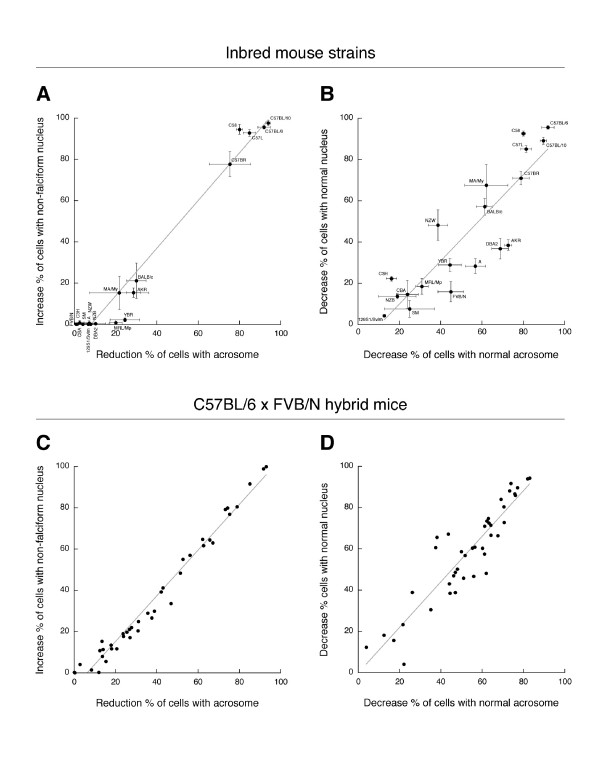
**Quantitation of effects of miglustat on morphology of spermatozoa from inbred mouse strains and interstrain hybrid mice**. Effects of miglustat administration on morphological features of cauda epididymal spermatozoa from (*A *and *B*) mice of various inbred strains (n = 2–3 per strain) and from (*C *and *D*) fourth-generation C57BL/6 × FVB/N hybrid mice. In (*A *and *C*) spermatozoa were scored for possession of an acrosome (present/absent), irrespective of acrosomal staining pattern, and for possession of a grossly abnormal non-falciform nucleus. In (*B *and *D*) spermatozoa were scored for acrosomal staining pattern (normal/abnormal), and for the normality of their nuclear morphology. Falciform nuclei (flat and curved) that deviated from the typical shape of spermatozoa from untreated mice were score as abnormal. In (*C *and *D*) data are presented from the hybrid mice that were most affected by miglustat, and from a representative number of hybrid mice that showed a lesser response. Each datapoint in (*A *and *B*) expresses the difference between the average score of drug-treated mice from one inbred strain and the average score of control mice of the same strain. Each datapoint in (*C *and *D*) expresses the difference between the score of one drug-treated hybrid mouse and the average score of untreated FVB/N mice. Data points were fitted by linear regression; the corresponding trend lines are displayed in grey (correlation coefficients in *A*, *B*, *C *and *D *were 0.97, 0.79, 0.98 and 0.84, respectively). Miglustat administration was at 150 mg/kg/day, except SM/J mice (15 mg/kg/day). At least 200 spermatozoa per mouse were scored for nuclear morphology and acrosomal staining.

In our previous studies [10-12], we used animals from the C57BL/6 strain. Here, we administered the drug to males of 18 additional strains other than C57BL/6. According to the genealogy of inbred strains of mice [18], 11 of the strains used were from the Swiss (FVB/N) and Castle (129S1/SvImJ, A/J, AKR/J, BALB/c, C3H/HeN, CBA/Ca, DBA/2, NZB, NZW and SM/J) families of strains, 6 from the C57 lineage (C57BL/10, C57BR, C57L, C58, MA/My and YBR), and one strain had a mixed background (MRL/Mp). After treating these mice with 150 mg/kg/day miglustat (a dose that renders C57BL/6 mice infertile), we examined the acrosomal status and nuclear shape of their cauda epididymal spermatozoa, by indirect immunofluorescence with an anti-acrosomal monoclonal antibody and a nuclear dye. The effects of the drug on sperm phenotype varied between mouse strains (Fig. 3). Because the vast majority of C57BL/6 spermatozoa are without an acrosome, and have a strongly abnormal (non-falciform) nuclear morphology after miglustat administration [10], we first scored the spermatozoa of the other strains for these features. This analysis divided the mouse strains into three groups. Firstly, five strains showed a major (>75%) drug-induced reduction in the percentage of acrosome-bearing spermatozoa and a high (>75%) increase in the proportion of sperm cells with non-falciform sperm nuclei (C57BL/6, C57BL/10, C57BR, C57L/J and C58/J, Fig. 4A). Secondly, three strains had moderate (15–30%) changes in both of these sperm parameters (BALB/c, AKR/J and MA/MyJ, Fig. 4A). Finally, in most strains the drug did not increase the proportion of spermatozoa with grossly misshapen nuclei, while the two most-affected strains of this third group (MRL/Mp and YBR) had a 20–25% reduction in the appearance of acrosomes (Fig. 4A). Thus the mice with the most perturbed sperm phenotype were from 5 closely related strains of the C57 family. The other two strains of this lineage were either moderately (MA/MyJ) or slightly (YBR) affected, according to the criteria applied.

Closer examination revealed that miglustat had a subtle, but discernible impact on the sperm phenotype of the Swiss/Castle strains. In many cases their spermatozoa did stain with the anti-acrosomal antibody, but displayed an irregular staining pattern, distinct from the typical crescent morphology (Fig. 3E, J and 3K). In addition, the nuclear morphology of the Swiss/Castle spermatozoa frequently deviated from the normal shape, while remaining falciform (i.e. flattened and to some extent curved, Fig. 3E, J and 3K). Thus, after administering miglustat to males of the 19 mouse strains, we also scored their sperm heads for the normality of the acrosomal staining pattern, and assessed the regularity of their nuclear morphology. In the Swiss/Castle strains the decrease in the percentage of spermatozoa with a normal acrosome ranged from 16 to 69%, while the percentage of cells with a typical falciform nuclear morphology was between 8 and 57% lower (Fig. 4B).

**Figure 5:**
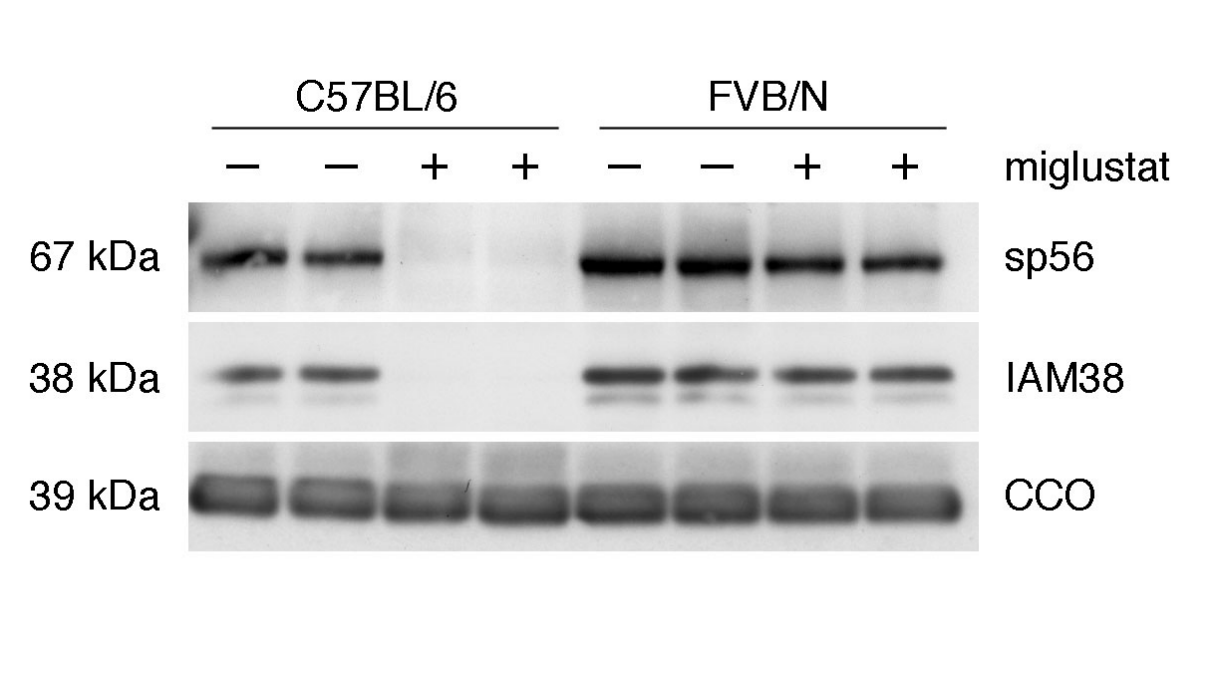
**Effects of miglustat on acrosomal proteins as detected by western blotting**. The impact of miglustat administration on the levels of the acrosomal proteins sp56 and IAM38 was assessed both in C57BL/6 and in FVB/N mice. Replicate western blots were prepared with lysates of epididymal spermatozoa and probed with antibodies against sp56, IAM38, or cytochrome C oxidase subunit I. Miglustat treatment was at 15 mg/kg/day.

The sperm phenotype of miglustat-treated FVB/N mice was further compared with that of the C57BL/6 strain. To complement the data on acrosome status obtained by immunostaining with monoclonal Mab18.6, we used western blotting to compare the levels of two other acrosomal components, a protein present in the acrosomal matrix (sp56, [17]) and a protein localized on the inner acrosomal membrane (IAM38, [16]). After administration of miglustat to C57BL6 mice neither sp56 nor IAM38 was detectable in their spermatozoa, while in drug-treated FVB/N mice the levels of these acrosomal proteins were comparable to those of untreated animals of this strain (Fig. 5). Thus the percentage of spermatozoa that stained with Mab18.6 after miglustat treatment (2.9% for C57BL/6 and 92% for FVB/N mice) correlated very well with the levels of sp56 and IAM38 as measured by western blotting.

**Figure 6:**
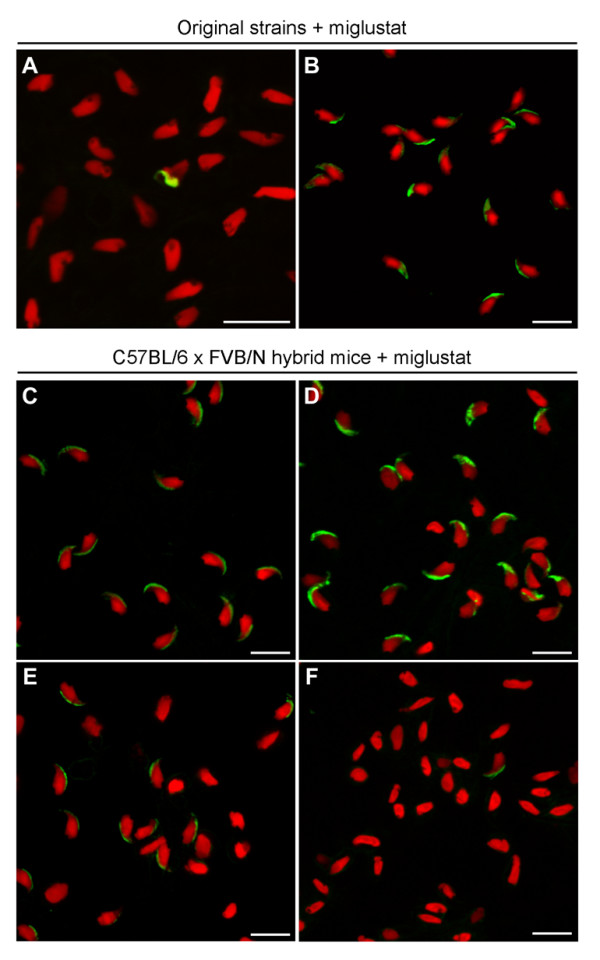
**Effects of miglustat on morphology of spermatozoa from interstrain hybrid mice**. Acrosomal and nuclear morphology of cauda epididymal spermatozoa from miglustat-treated (*A*) C57BL/6 (15 mg/kg/day) and (*B*) FVB/N mice (600 mg/kg/day), and from (*C*, *D*, *E *and *F*) fourth-generation C57BL/6 × FVB/N hybrid mice (15 mg/kg/day). These drug-treated hybrid mice differed in their proportion of non-falciform acrosome-less spermatozoa. This proportion increases from *C *to *F*. Acrosomes were stained with the monoclonal antibody Mab18.6 (green), and nuclei with propidium iodide (red). Bar = 10 μm.

Furthermore, after treatment of FVB/N mice with a higher dose of miglustat (600 mg/kg/day) the appearance of their spermatozoa was comparable to that seen at 150 mg/kg/day (Fig. 6B and Fig. 3E, respectively). Both at 150 and 600 mg/kg/day the drug-induced abnormalities were limited to mild nuclear and acrosomal irregularities. The percentages of spermatozoa with these defects did not differ significantly between the lower and higher drug dose (data not shown).

### Miglustat has a variable impact on the fertility of male mice

To establish whether the miglustat-induced alterations in the sperm phenotypes from the Swiss/Castle mice were relevant for their fertility, drug-treated males (150 mg/kg/day) from a number of these strains were assessed in a natural mating test. The strains used in this test were selected to represent the extent of variation in the responses to miglustat among the Swiss/Castle strains (Fig. 4B), from slight (129S1/SvlmJ), moderate (FVB/N), to more profoundly affected (AKR/J and DBA/2J). In addition, the moderately impacted C57 strain MA/MyJ (Fig. 4A) was also tested. In contrast to miglustat-treated C57BL/6 mice, the fertility of the 129S1/SvlmJ, FVB/N and DBA/2J males was unchanged (Table 1). Miglustat treatment reduced the size of litters sired by the AKR/J and MA/MyJ males, but this was not statistically significant (Table 1). Thus the considerable increase (69%) in the percentage of spermatozoa with a misshapen acrosome, in combination with moderately more (37%) spermatozoa with an abnormal falciform nuclear morphology, did not impair the fertility or size of litters sired by male mice, as seen in the DBA/2J strain. Similarly, with a 29% increase in spermatozoa without an acrosome and 16% of sperm cells with a severely abnormal (non-falciform) nuclear shape, the AKR/J were not infertile. Therefore, whereas miglustat affected the sperm phenotype in many mouse strains (Fig. 3 and Fig. 4A and 4B), this did not result in infertility in most cases (Table 1). Clearly, drug-induced infertility (as seen in C57BL/6 mice) was associated with a major absence of acrosomes and high proportion of grossly aberrant sperm nuclei.

### Miglustat levels in serum in mouse strains and rabbits

The variation in the effects of miglustat among rabbits and different mouse strains could be due to unequal serum levels, which, in turn, may be a reflection of variability in the rate of renal drug excretion. Therefore, miglustat levels in serum were determined by LC/MS. Although administration of 15 mg/kg/day miglustat s.c. via mini osmotic pumps rendered C57BL/6 but not FVB/N mice infertile, the drug treatment generated a similar miglustat level in the serum of C57BL/6 and in FVB/N mice (Table 2). In rabbits the serum level was determined at the highest dose used (50 mg/kg/day), at which the animals were fertile. The serum level found in rabbits exceeded the level in C57BL/6 mice 17-fold (Table 2). Thus, the serum concentration of miglustat did not correlate with the fertility status of the mice and rabbits.

**Table 2:** Serum levels of miglustat in selected strains/species.

Species	Mouse		Rabbit
Strain	C57BL/6	FVB/N	Russen-kaninchen

Dose (mg/kg/day)	15	15	50
Serum level (μM)	0.49 ± 0.26	0.61 ± 0.17	8.39 ± 5.38
Fertility (fertile/total)	0/6	6/6	5/5

Miglustat was administered to both strains of mice via a miniosmotic pump and miglustat was given to rabbits via their food. Male mice were caged with 2 single females subsequently at week 5 and 6. Male rabbits were mated to one female each. Serum levels were determined from blood serum by means of LC/MS. Data are expressed as means ± SD.

### Genetic basis of the susceptibility to miglustat

Having observed that the consequences of miglustat administration varied between mouse strains, we reasoned that the differential response to the drug possibly has a genetic basis. Therefore, we generated small numbers of second-generation interstrain hybrid mice from the C57BL/6 and FVB/N strains. Eight F2 hybrids were obtained from C57BL/6 males and FVB/N females, and 6 F2 hybrids were bred from FVB/N males and C57BL/6 females. In each of these two groups of F2 hybrid mice one animal displayed the C57 drug-induced sperm phenotype and one animal had a phenotype that was intermediate between the two original strains. The remainder of the F2 hybrids responded to miglustat administration in the same way as FVB/N mice (data not shown). Thus, the high sensitivity to the imino sugar (resulting in the production of acrosome-less non-falciform abnormal spermatozoa) had both a maternal and a paternal inheritance.

To further investigate the genetic basis of the imino sugar responsiveness, we generated over 200 fourth-generation hybrid mice from C57BL/6 males and FVB/N females. These animals were treated with 15 mg/kg/day of miglustat, and their epididymal spermatozoa were examined and scored for the acrosomal and nuclear parameters described above. Over 70% of the hybrid mice responded to miglustat administration in a similar fashion as the FVB/N mice, while only 3 of them behaved like a C57BL/6 mouse. However, the remainder of the interstrain hybrid mice showed a range of effects, from low to very high percentages of non-falciform dysmorphic spermatozoa without acrosomes (Fig. 4C and Fig. 6C–F). Among the hybrid mice were a number of animals with an intermediate drug-induced phenotype (between 30 and 70% reduction in acrosome presence, and between 20 and 70% increase in non-falciform abnormal nuclei, Fig. 4C). Such a phenotype was not seen in any of the inbred mouse strains after miglustat treatment (Fig. 4C vs. 4A). Similar to the inbred strains, the epididymal spermatozoa from the interstrain hybrids also displayed irregular acrosomal structures and atypical falciform nuclei (Fig. 4D and Fig. 6C–F).

## Discussion

We have evaluated the reproductive effects of miglustat in rabbits and in various strains of the C57 and Swiss/Castle lineages of inbred mice. In contrast to miglustat-treated C57 mice, fertility was not affected in the other mouse strains and the rabbit strain that have been studied. The contraceptive effect of miglustat seems therefore to be specific to the C57 strains.

In mice, the most profound effects of miglustat on the shape of sperm nuclei and on the presence of acrosomes on sperm heads were found in strains of the C57 family (the majority of sperm nuclei non-falciform dysmorphic, most spermatozoa without an acrosome), correlating closely with the contraceptive action in C57BL/6 mice. A milder category of sperm aberrations after miglustat treatment was found in most strains of the Swiss/Castle lineages (low to moderate frequencies of mild morphological abnormalities of falciform nuclei, most spermatozoa with acrosomes, a fraction of them with imperfections or aberrations). Clearly, this type of spermatozoal abnormalities is not of a severity that impairs the fertility of the animals, as the drug-treated 129S1/SvImJ, FVB/N and DBA/2 males had normal pregnancy rates in mating tests. We also observed an intermediate level of sperm aberrations, in miglustat-treated BALB/c, Ma/MyJ and AKR/J mice (a minority of spermatozoa with non-falciform nuclei, without acrosomes). This level of miglustat-induced spermatozoal abnormalities was not associated with infertility in the drug-treated Ma/MyJ and AKR/J mice. Finally, in one rabbit strain miglustat administration did not result in any observable changes in sperm phenotype.

What therefore underlies the differences in the effect of miglustat between various mouse strains and between species? One possibility is that the males of the Swiss/Castle strains and the rabbits could be less sensitive to the drug. That would imply that a higher dose of miglustat should result in a more severe phenotype in these strains and in rabbits. We have investigated this in FVB/N mice, by administering 150 and 600 mg/kg/day of miglustat. We found the same effects in males of this strain at both doses of the drug, not a C57-style sperm phenotype at the higher dose. This suggests that it is unlikely that the disparity in the imino sugar-response between Swiss/Castle and C57 strains is due to a lower drug sensitivity of the former strains, rather that the consequences of miglustat in these strains are qualitatively different. Secondly, the differences in reproductive outcome of miglustat treatment observed between mouse strains and species could be due to differential pharmacokinetics of the imino sugar (e.g. variations in the rate of renal excretion), resulting in different serum concentrations when administered at the same dose of drug. Clearly, this was not the case as serum levels were the same or even higher in the non-responding FVB/N strain (0.6 μM) and in rabbits (8.4 μM), compared to the highly responding C57BL/6 mice (0.5 μM). In men, the miglustat level was >4 μM in serum and 8 μM in seminal plasma [13]

Recently, it has been speculated that the primary target of miglustat is β-glucosidase 2 (GBA2), because a deficiency in GBA2 results in the production of abnormal spermatozoa [19,20], and because GBA2 can be inhibited by compounds that are chemically similar to miglustat [21]. In addition, GBA2 has been identified as being responsible for the non-lysosomal glucosylceramidase activity [22], which can be inhibited by miglustat [5]. To date it has not been reported whether miglustat inhibits GBA2 *in vivo*. Nevertheless, it is conceivable that the interaction of miglustat with its primary target (possibly GBA2) is the first step in a cascade of events that brings about the dramatic effects of miglustat on spermiogenesis in C57 strains. This cascade including downstream components needs to be considered when investigating the basis of the diversity in the reproductive consequences of miglustat in the various mouse strains and between species. Possibly, the direct effect of miglustat on the primary drug target, or its indirect impact on a component of the downstream pathway is strain/species-dependent. Studies are currently in progress focusing on the *in vivo *biochemical consequences of miglustat administration, with particular emphasis on the glycosphingolipid pathway.

Our studies with the C57BL/6 × FVB/N interstrain hybrid mice provide an indication of the genetics of the sensitivity to miglustat. Whereas the majority of the fourth-generation hybrid mice responded to miglustat in a similar fashion as FVB/N mice, only very few displayed the C57BL/6 phenotype. About one-third of the interstrain hybrid mice simultaneously produced spermatozoa with an acrosome and a falciform nuclear shape, as well as acrosome-deficient spermatozoa with grossly aberrant nuclei. Remarkably, these two types of spermatozoa were produced in various proportions that had not been seen in either of the parental strains. Clearly, the sensitivity of spermatogenesis for miglustat is not inherited in a Mendelian fashion. Rather, the reproductive impact of miglustat, expressed as the percentage of acrosome-less spermatozoa with a non-falciform nuclear shape, appears to be a quantitative trait. It is therefore likely that multiple genes contribute to the sensitivity of spermatogenesis to miglustat. Similar observations have been made in the study of the susceptibility to lung injury by ozone. Mice of the A/J strain are susceptible in this respect, while C57BL/6 mice are resistant [23]. Recombinant inbred strains generated from these two strains displayed many intermediate levels of ozone susceptibility, in a continuous range, indicating a multigenic trait [24].

The observation that the effect of a drug, a toxicant, or gene ablation is dependent on genetic background is not uncommon, and has been seen in many fields, including reproductive biology. For example, low-dose cadmium causes necrosis in the seminiferous epithelium in DBA/2, but not in C57BL/6 mice [25]. The resistance to this heavy metal is an autosomal recessive trait that is inherited in a Mendelian fashion [26]. In three distinct lines of knockout mice, the deficiency results in male sterility in a 129/Sv background, but not in mixed C57BL/6 × 129/Sv animals [27-29]. Germ cell depletion 2 (*gcd2*), a chemically induced recessive mutation, affects almost all males when homozygously present in a CAST/Ei mice, but only a minority of mice against a C57BL/6 background [30]. Also, the male infertility, seen in the leptin-deficient *ob*/*ob *mice that are on a C57BL/6 background, is rescued when the deficiency is bred into the BALB/c strain [31,32]. In addition, experimentally induced cryptorchidism has a negative impact on spermatogenesis in most strains, but not in AKR/N and MRL/Mp mice [33].

It will be interesting to determine which genetic factors determine the susceptibility to miglustat, not the least because the capacity of the drug to specifically interfere in acrosome biogenesis is exceptional (even when manifest in only a limited number of mouse strains). Various approaches can be followed in this endeavour, starting with a genomic evaluation of the panels of the C57BL/6 × FVB/N interstrain hybrid mice and of the 19 mouse strains, in which the compound has been tested. These studies are currently in progress.

## Competing interests

WB, MF, UV, EL and UG are employees of Schering AG, Germany. FMP and ACS are in receipt of a research grant from Schering AG, Germany.

## Authors' contributions

WB participated in the conception and design of the study, in the execution of experiments, analysis of experimental data, preparation of the data for publication, and in the writing of the manuscript. CMW participated in the execution of experiments, in the analysis of experimental data. MF, UV, EL and UG participated in the conception and design of the study and revised the manuscript critically. SB performed and analyzed western blotting experiments. FMP participated in the design of the study, and in writing of the manuscript. ACS participated in the conception and design of the study, in the execution of experiments, analysis of experimental data, preparation of the data for publication, and in the writing of the manuscript. All authors read and approved the final manuscript.
